# Methadone Prescribing and Overdose and the Association with Medicaid Preferred Drug List Policies — United States, 2007–2014

**DOI:** 10.15585/mmwr.mm6612a2

**Published:** 2017-03-31

**Authors:** Mark Faul, Michele Bohm, Caleb Alexander

**Affiliations:** ^1^National Center for Injury Prevention, CDC; ^2^Center for Drug Safety and Effectiveness and Department of Epidemiology, Johns Hopkins Bloomberg School of Public Health, Baltimore, Maryland.

Drug overdose is a leading cause of injury death in the United States; 47,055 fatal drug overdoses were reported in 2014, a 6.5% increase from the previous year (*1*), driven by opioid use disorder (*2*,*3*). Methadone is an opioid prescribed for pain management and is also provided through opioid treatment programs to treat opioid use disorders. Because methadone might remain in a person’s system long after the pain-relieving benefits have been exhausted, it can cause slow or shallow breathing and dangerous changes in heartbeat that might not be perceived by the patient (*4*,*5*). In December 2006, the Food and Drug Administration issued a Public Health Advisory that alerted health care professionals to reports of death and life-threatening adverse events, such as respiratory depression and cardiac arrhythmias, in patients receiving methadone (*4*); in January 2008, a voluntary manufacturer restriction limited distribution of the 40 mg formulation of methadone.* CDC analyzed state mortality and health care data and preferred drug list (PDL) policies to 1) compare the percentage of deaths involving methadone with the rate of prescribing methadone for pain, 2) characterize variation in methadone prescribing among payers and states, and 3) assess whether an association existed between state Medicaid reimbursement PDL policies and methadone overdose rates. The analyses found that, from 2007 to 2014, large declines in methadone-related overdose deaths occurred. Prescriptions for methadone accounted for 0.85% of all opioid prescriptions for pain in the commercially insured population and 1.1% in the Medicaid population. In addition, an association was observed between Medicaid PDLs requiring prior authorization for methadone and lower rates of methadone overdose among Medicaid enrollees. PDL policies requiring prior authorization might help to reduce the number of methadone overdoses.

To calculate drug overdose deaths and corresponding mortality rates, National Vital Statistics System Multiple Cause of Death mortality files (*6*) and bridged U.S. Census data for the period 1999–2014 were analyzed. To assess whether methadone prescribing in particular is higher among Medicaid enrollees, Truven Health’s MarketScan Commercial Claims and Encounters (CCE) and Medicaid multistate databases for 2014 were used to compare outpatient methadone prescribing rates for commercially insured populations with Medicaid populations.^†^ The CCE database represents enrollees who are typically covered through large private employers and state governments, enabling creation of a regionally distributed convenience sample of privately insured persons.

To explore whether the observed decline in methadone overdose deaths from 2007 to 2014 was associated with Medicaid methadone reimbursement policies aimed at reducing methadone prescribing, methadone overdoses (including fatal and nonfatal overdoses) were examined. Some states use a PDL, a formal published list of specific prescription drug products by brand and generic name, listed as “preferred.” Nonpreferred products are available for payment or reimbursement only after obtaining prior authorization for the particular patient and product. Prescribing drugs from the preferred list makes the approval process less cumbersome and facilitates faster reimbursement. To determine whether a state’s policy was associated with higher methadone morbidity or mortality, 2012 and 2013 emergency department and inpatient data from the Health Care Utilization Project (HCUP) (*7*) from three states (Florida, North Carolina, and South Carolina) were analyzed. State selection was based on geographic proximity (to maximize population similarities), variation in state PDL policies, and data availability. For each state, it was determined whether the PDL included methadone for pain; usually a prescriber does not have to obtain prior approval for use of a PDL drug to obtain reimbursement.

The three selected states confirmed the status of methadone for pain on their PDLs with the Centers for Medicare & Medicaid Services. During 2012–2013, Florida listed methadone as a preferred drug on its PDL. North Carolina gave methadone a preferred status without listing it on its PDL (Centers for Medicare & Medicaid Services, unpublished data, 2017), and South Carolina did not include methadone as a preferred drug. HCUP data (*7*) were used to calculate rates of methadone overdose by state for Medicaid enrollees; administrative billing codes from the *International Classification of Diseases, Ninth Version, Clinical Modification* (ICD-9-CM) were used to identify methadone overdose cases (965.02 [poisoning by methadone] and external cause code E8501 [accidental poisoning by methadone]). Fatal and nonfatal overdose cases were identified in both state-specific emergency department and inpatient data. Medicaid enrollee eligibility population within each state was provided by HCUP and used for population denominators. Univariate analysis of variance (ANOVA) with F-test was used to analyze methadone rates of overdose among Medicaid-reimbursed patients in the three selected states. Differences with p values <0.05 were considered statistically significant. All analyses were performed using statistical software.

## Trends in Methadone Mortality

**FIGURE 1:**
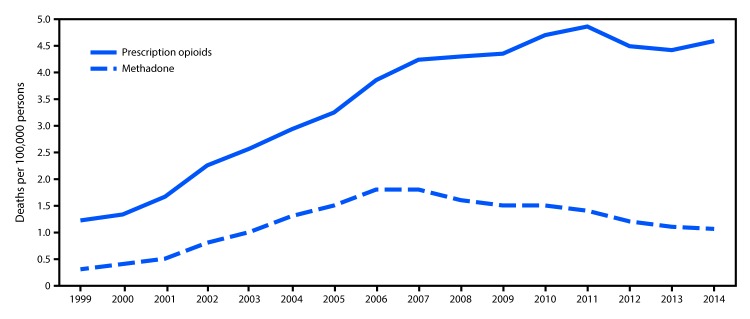
Rate of deaths from prescription opioid overdose overall* and from methadone overdose — United States, 1999–2014 * Prescription opioid deaths include those involving natural and semisynthetic opioids and methadone.

From 1999 to 2014, the overall prescription opioid overdose death rate (involving natural and semisynthetic opioids and methadone) increased 300%, from 1.2 persons per 100,000 population (3,442 persons) in 1999 to 4.6 (14,838) in 2014 (Figure 1). The rate of methadone overdose deaths increased 600%, from 0.3 persons per 100,000 in 1999 (784) to 1.8 in 2006 (5,406), was stable in 2007 (5,518), and then declined 39% to 1.1 (3,400) in 2014.

## Methadone Prescriptions Among Medicaid Enrollees

**FIGURE 2:**
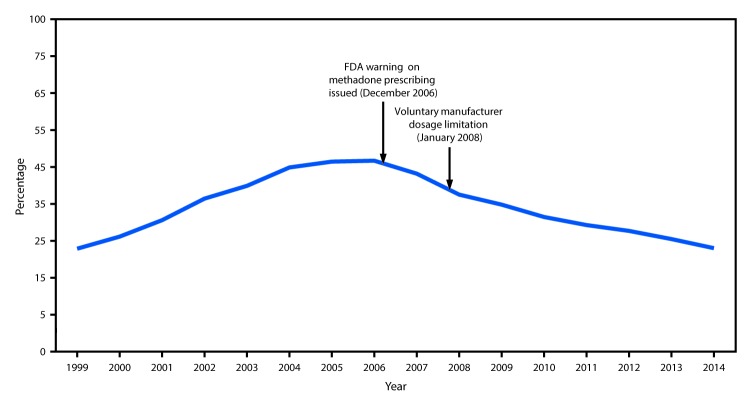
Percentage of prescription opioid overdose deaths involving methadone — United States, 1999–2014 **Abbreviation:** FDA = Food and Drug Administration.

Prescriptions for methadone accounted for 0.85% (weighted) of all opioid prescriptions for pain in the commercially insured population and 1.1% in the Medicaid population, indicating that methadone prescribing for pain constituted a small proportion of opioid analgesic use. However, although methadone accounted for approximately 1% of all opioid prescriptions, overall methadone-related deaths accounted for 22.9% of all opioid-related mortality in 2014 (Figure 2). Among 20.9 million CCE enrollees and 6.8 million continuously enrolled Medicaid enrollees, the 2014 methadone prescribing rate among Medicaid enrollees (9.33 per 1,000 enrollees) was nearly twice that of CCE enrollees (4.85 per 1,000 enrollees).

## Association Between State Medicaid PDLs and Overdose Deaths

**FIGURE 3:**
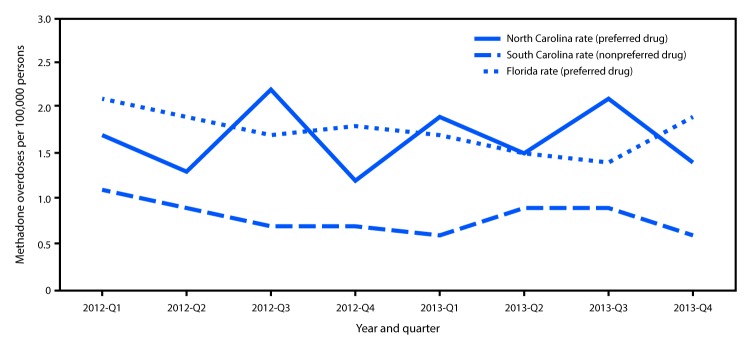
Methadone overdose rates among Medicaid enrollees, by year and quarter — Florida, North Carolina, and South Carolina, 2012–2013

The rates of fatal and nonfatal methadone overdose among Medicaid enrollees in Florida (1.75 per 100,000 persons; 95% confidence interval [CI] = 1.57–1.94) and North Carolina (1.67, CI = 1.35–1.98), the two analyzed states that included methadone as a preferred drug, were significantly higher than those in South Carolina (0.81, CI = 0.65–0.96), which did not include methadone as preferred (Figure 3). The rate in South Carolina was significantly lower than the rates in North Carolina (F = 39.89, p<0.001) and Florida (F = 48.49, p<0.001). The rates of methadone overdose in North Carolina and Florida were similar (F = 0.42, p<0.525). Whereas there were large differences among the states in methadone overdose rates, the overall opioid overdose death rates in 2013 were similar for Florida (13.2 per 100,000 persons), North Carolina (12.9), and South Carolina (13.0) (*1*).

## Discussion

Drug overdose deaths involving methadone peaked in 2006 and 2007, then declined 39% by 2014. Despite this decline, however, methadone continues to account for nearly one in four prescription opioid-related deaths. Although this study was not designed to assess causal inference, the peak inflection point in 2007 occurred shortly after the December 2006 issuance of the Food and Drug Administration’s Public Health Advisory on prescribing methadone that linked reports of respiratory depression and cardiac arrhythmias with the possibility of unintentional overdoses, drug interactions, or cardiac toxicity (*4*). The voluntary manufacturer restriction limiting the 40 mg formulation of methadone in 2008 likely also contributed to declines in methadone overdose death rates (*8*).

Given that methadone prescribing rates are higher among persons enrolled in Medicaid, strategies to reduce methadone prescribing among persons in this population might further reduce injuries and deaths from methadone. Focusing on the differences between state PDLs, a comparative exploratory analysis of states with different methadone drug utilization management policies found an association between a state’s internal PDL policy and methadone overdose rates. If confirmed by additional studies, other states could consider Medicaid drug utilization management strategies such as PDL placement among other evidence-based strategies to reduce injuries and deaths associated with methadone.^§^ Other pharmacy management strategies (e.g., prior authorization, quantity limits, and retrospective drug utilization review), as well as adherence to clinical prescribing guidelines and the increased deployment of prescription drug monitoring programs, might also help to optimize the benefit of methadone. Many of these approaches might also be applicable to private insurers.

The findings in this report are subject to at least two limitations. First, the analysis of mortality and morbidity data included all methadone overdoses. Because methadone is prescribed for pain and also to treat opioid use disorders in community-based opioid treatment programs, there is no definitive way to determine the source of methadone contributing to an injury or death. However, because methadone prescribed to treat opioid use disorders is tightly regulated (including an extra set of special standards) (*9*), the preponderance of methadone-associated morbidity and mortality likely arises from its use for pain. Second, findings from the policy analysis of PDL and overdose rates are exploratory in nature, and there are many potential determinants of methadone-related overdose rates beyond PDL policies. For example, South Carolina reported in its fiscal year 2013 Medicaid Drug Utilization Annual Report that it had implemented other drug utilization management strategies, such as requiring pain management providers to be certified and a process to identify prescribers not authorized to prescribe controlled drugs, whereas North Carolina and Florida did not have these policies at that time.

Amid a growing epidemic of deaths with widespread overuse of prescription opioids, understanding the successful strategies for the reduction in methadone overdose are important and might serve as a model for future positive outcomes involving other opioid drugs. Options for reducing future opioid morbidity and mortality include implementing multiple drug utilization management policies that are consistent with PDL practices and the CDC *Guideline for Prescribing Opioids for Chronic Pain* (*10*), which recommends that methadone should not be the first choice for an extended-release/long acting opioid.

**Summary**

**What is already known about this topic?**

It is important that prescribing methadone as a pain medication is done carefully. In 2006, the Food and Drug Administration issued a public health advisory regarding health risks associated with prescribing methadone.

**What is added by this report?**

Methadone accounted for approximately 1% of all opioids prescribed for pain but accounted for approximately 23% of all prescription opioid deaths in 2014. State drug management practices and reimbursement policies can affect methadone prescribing practices and, in turn, might reduce methadone overdose rates within a state.

**What are the implications for public health practice?**

Drug utilization management policies that reduce the use of risky opioids such as methadone might reduce opioid-related morbidity and mortality. This evidence of decreases in methadone overdoses and use of preferred drug list policies could serve as a model for future decreases in other specific opioid drug-related mortality.

## Footnotes

* https://www.deadiversion.usdoj.gov/drug_chem_info/methadone/methadone.pdf.

† https://marketscan.truvenhealth.com/marketscanportal/.

§ https://www.medicaid.gov/federal-policy-guidance/downloads/cib-02-02-16.pdf.
